# Geographic Variation in Late-Stage Cervical Cancer Diagnosis

**DOI:** 10.1001/jamanetworkopen.2023.43152

**Published:** 2023-11-13

**Authors:** Itunu O. Sokale, Aaron P. Thrift, Jane Montealegre, Victor Adekanmbi, Onyema G. Chido-Amajuoyi, Ann Amuta, Lorraine R. Reitzel, Abiodun O. Oluyomi

**Affiliations:** 1 Department of Medicine, Section of Epidemiology and Population Sciences, Baylor College of Medicine, Houston, Texas; 2 Dan L. Duncan Comprehensive Cancer Center, Baylor College of Medicine, Houston, Texas; 3 Department of Behavioral Science, The University of Texas MD Anderson Cancer Center, Houston; 4 Department of Obstetrics and Gynecology, University of Texas Medical Branch, Galveston; 5 Department of Internal Medicine, Texas A&M School of Medicine/Christus Health, Longview; 6 School of Health Promotion and Kinesiology, Texas Woman’s University, Denton; 7 Department of Health Disparities Research, The University of Texas MD Anderson Cancer Center, Houston

## Abstract

**Importance:**

There are stark disparities in cervical cancer burden in the United States, notably by race and ethnicity and geography. Late-stage diagnosis is an indicator of inadequate access to and utilization of screening.

**Objective:**

To identify geospatial clusters of late-stage cervical cancer at time of diagnosis in Texas.

**Design, Setting, and Participants:**

This population-based cross-sectional study used incident cervical cancer data from the Texas Cancer Registry from 2014 to 2018 of female patients aged 18 years or older. Late-stage cervical cancer cases were geocoded at the census tract level (n = 5265) using their residential coordinates (latitude and longitude) at the time of diagnosis. Statistical analysis was performed from April to September 2023.

**Exposures:**

Census tract of residence at diagnosis.

**Main Outcome and Measures:**

Late-stage cervical cancer diagnosis (ie, cases classified by the National Cancer Institute Surveillance, Epidemiology and End Results summary stages 2 to 4 [regional spread] or 7 [distant metastasis]). A Poisson probability-based model of the SaTScan purely spatial scan statistics was applied at the census tract–level to identify geographic clusters of higher (hot spots) or lower (cold spots) proportions than expected of late-stage cervical cancer diagnosis and adjusted for age.

**Results:**

Among a total of 6484 female patients with incident cervical cancer cases (mean [SD] age, 48.7 [14.7] years), 2300 (35.5%) were Hispanic, 798 (12.3%) were non-Hispanic Black, 3090 (47.6%) were non-Hispanic White, and 296 (4.6%) were other race or ethnicity. Of the 6484 patients, 2892 with late-stage diagnosis (mean [SD] age, 51.8 [14.4] years were analyzed. Among patients with late-stage diagnosis, 1069 (37.0%) were Hispanic, 417 (14.4%) were non-Hispanic Black, 1307 (45.2%) were non-Hispanic White, and 99 (3.4%) were other race or ethnicity. SaTScan spatial analysis identified 7 statistically significant clusters of late-stage cervical cancer diagnosis in Texas, of which 4 were hot spots and 3 were cold spots. Hot spots included 1128 census tracts, predominantly in the South Texas Plains, Gulf Coast, and Prairies and Lakes (North Texas) regions. Of the 2892 patients with late-stage cervical cancer, 880 (30.4%) were observed within hot spots. Census tract–level comparison of characteristics of clusters suggested that hot spots differed significantly from cold spots and the rest of Texas by proportions of racial and ethnic groups, non–US born persons, and socioeconomic status.

**Conclusions and Relevance:**

In this cross-sectional study examining geospatial clusters of late-stage cervical cancer diagnosis, place-based disparities were found in late-stage cervical cancer diagnosis in Texas. These findings suggest that these communities may benefit from aggressive cervical cancer interventions.

## Key Points

**Question:**

Were there spatial variations in late-stage cervical cancer diagnosed in Texas from 2014 to 2018, and how did characteristics of clusters of late-stage cervical cancer compare with nonclusters?

**Findings:**

In this population-based cross-sectional study of 6484 female patients with incident cervical cancer in Texas, including 2892 with late-stage diagnosis, 4 significant hot spots of late-stage cervical cancer diagnoses were identified. Hot spots were located in the South Texas Plains, Gulf Coast (Houston), and Prairies and Lakes (North Texas) regions; and they had higher than national rates of individuals who were Hispanic, non–US born, and socioeconomically disadvantaged.

**Meaning:**

These findings suggest that additional funding should be allocated for area-specific interventions in Texas to reduce cervical cancer disparities in hot spots of late diagnosis.

## Teaser

This cross-sectional study investigates geospatial clusters of late-stage cervical cancer at time of diagnosis in Texas.

## Introduction

Despite advancements in cervical cancer prevention and diagnosis, more than 340 000 preventable deaths from cervical cancer occurred worldwide in 2020.^1,2^ In the US, approximately 14 000 women are newly diagnosed with cervical cancer yearly, and about 4000 women die of advanced cervical cancer annually.^3^ From 2012 to 2016, Texas had a higher cervical cancer incidence rate (9.2 cases per 100 000 women) compared with the US national rate (7.6 per 100 000 women).^4^

Widespread population-based cervical cancer screening led to declining cervical cancer incidence rates in high-income countries with organized screening programs.^5^ Cervical cancer cases detected early through screening or diagnostic testing are easier to treat and have better 5-year relative survival (92%) than those diagnosed with regional or distant spread (59% for regional and 17% for distant spread).^6^ Late-stage cervical cancer (LCC) diagnosis persists and is rising in the US.^7^ This is likely reflective of inequitable access to screening, especially among women from minoritized racial and ethnic groups and rural women,^8,9,10,11^ Compared with non-Hispanic White women, non-Hispanic Black and Hispanic women in the US have significantly higher incidence and mortality rates of cervical cancer.^12,13^ Cervical cancer screening rates in Texas remain suboptimal.^14^ In addition, cervical cancer incidence is higher in Texas compared with the rest of the US, and within Texas, some regions such as Northeast Texas have even higher incidence burden compared with the rest of the state.^15^ In Texas, cervical cancer health disparities have been described in terms of stage at diagnosis, race and ethnicity, and rurality.^16^

Spatial analysis can identify places with significant differences in relative risk of diseases. Previous studies have conducted spatial analyses to detect geographic clusters in incidence, mortality, and survival from various diseases, including cancers, globally.^17,18,19,20,21,22,23^ If identified, LCC clusters may be targeted for more intervention resources to enhance screening and early detection. However, studies objectively demonstrating geographical clusters of delayed cervical cancer diagnosis with racial and ethnic distribution in the US overall and in Texas specifically, are scarce.^24,25^ Identifying LCC diagnosis concentration may inform strategic and effective interventions to improve early diagnosis in Texas and reduce the burden of cervical cancer. In this study, we examined spatial clusters of LCC at diagnosis in Texas from 2014 to 2018 and compared the population characteristics of census tracts in clusters with higher- and lower-than-expected proportions of LCC cases, as well as with the rest of the state of Texas.

## Methods

### Study Design, Data Source, Study Population

In this cross-sectional study, incident cervical cancer cases were obtained from the Texas Cancer Registry (TCR) from 2014 to 2018. TCR is a statewide population-based registry of all cancers diagnosed in Texas.^4^ The registry has case completion rates greater than 95% and joined the National Cancer Institute Surveillance, Epidemiology and End Results (SEER) Program in 2021. TCR data include demographics (race and ethnicity are collected via medical records as either self-reported or inferred from a clinician), clinical characteristics at diagnosis, and treatment information for each cancer case. Primary site and histology are coded to the *International Classification of Diseases for Oncology, Third Edition (ICD-0-3)* and stage at diagnosis was based on the SEER summary stage.^26^ The institutional review boards of Baylor College of Medicine and Texas Department of State Health Services reviewed and approved this study. We followed the Strengthening the Reporting of Observational Studies in Epidemiology (STROBE) reporting guideline.

We retrieved all primary cervical cancer cases (*ICD-0-3* C530, C531, C538, and C539) in the TCR database, diagnosed among female patients aged 18 years or older living in Texas at the time of diagnosis from January 2014 to December 2018 (n = 6602). We excluded duplicate cases (n = 2), those ascertained by autopsy reports (n = 0) or death certificates (n = 88), missing or nonvalid geographic identification numbers (n = 28). The final sample size of all patients with diagnosed cervical cancer included in the present study was 6484, including 2732 (42.1%) with early-stage (SEER summary stage 0 [in situ, intraepithelial, noninvasive]) and 1 [localized]), 2892 (44.6%) with late-stage (SEER summary stages 2-4 [regional spread] and 7 [distant spread]), and 860 (13.3%) with unstaged (SEER summary stage 9 [missing or unknown stage]) cervical cancer at diagnosis.

### Variables and Measures

#### Study Outcome and Covariates

The variable of interest for geospatial analyses study was LCC diagnosis. For individual-level descriptive analysis, we included age at diagnosis, race and ethnicity, payer at diagnosis, and diagnosis year. For the census tract-level descriptive analysis, we included median age, race and ethnicity, nativity, English language proficiency, educational attainment, employment status for individuals 16 years or older, median household income, and health insurance for female patients aged 18 years or over. We combined race and ethnicity variables and recoded as Hispanic, non-Hispanic Black, non-Hispanic White, and other. Other race and ethnicity included patients who identified as races other than White and Black (eg, American Indian or Alaska Native or Asian).

#### Data Assembly Considerations

The data assembly for the analyses involved multiple processes. To conduct the cluster analyses, we prepared 3 input files (case, population, and coordinate files) for the SaTScan analyses using census tract as the unit of analysis.^27^ A census tract is a small, homogenous, and relatively permanent statistical subdivision of a county, often used to represent neighborhood-level factors in epidemiologic or health services studies in the US. Usually, a census tract consists of approximately 4000 residents or 1600 housing units.^28^

#### Case File

We used the TCR-provided longitude (X) and latitude (Y) geographical coordinates representing each residential address of patients with LCC at the time of diagnosis. The coordinates were geocoded in ArcGIS Pro version 3.0 (Esri) and linked to the census tracts that contained them by overlaying the referenced location for each case with the corresponding boundary from the 2010 US Census Bureau TIGER/Line census tract shapefile. We then summed the counts of LCC cases from 2014 to 2018 in each census tract, and then by age categories (18-34 years, 35-64 years, 65 years or older) for each census tract.

#### Population File

We retrieved population data of female patients aged 18 years or older (the corresponding at-risk population) from the American Community Survey (ACS) 5-year (2014-2018) estimates matching their geographic identification numbers to those of LCC cases across the 5265 Texas census tracts. We then summed the counts of the at-risk population in each census tract, and then by age groups (18-34 years, 35-64 years, and 65 years or older) for each census tract.

#### Coordinates File

With the coordinates file, we specified the census tract location identification numbers (IDs), longitude (X), and latitude (Y) for the LCC cases and population. Lastly, to compare census tract–level population characteristics of the SaTScan-identified hot spots vs cold spots vs rest of the state, we used census tract–level demographic variables listed earlier from the 2014 to 2018 ACS 5-year estimate as proxy for individual-level measurement of socioeconomic status, health care access and use, as in previous studies.^23,24,29^ ACS data on race and ethnicity are collected through self-reported surveys.

### Statistical Analysis

Statistical analysis was performed from April to September 2023. To identify and analyze geographic areas with statistically significantly higher or lower proportions (spatial clusters) of LCC cases at diagnosis in Texas from the year 2014 to 2018, we applied a Poisson-based purely spatial scan statistical analyses in SaTScan software version 10.1 (Kulldorff).^27^ SaTScan is a widely used cluster-detecting software in various fields, including epidemiology, where it is often used to describe the geographical variations of diseases.^30^ The Poisson probability model was selected because we have count data of a relatively rare disease in a background population from which the cases arise, based on the null hypothesis that LCC cases are independent of each other. The Poisson model calculates the expected number of cases (*E*) in each location under the null hypothesis using indirect standardization: *E[c] = p × C / P* (where *c* is the observed number of cases, *p* is the population in the location, *C* total number of cases, and *P* is population). In addition, the Poisson model allows for covariate adjustment by specifying covariates in the input files: *E[c] = ∑_i_ E[c_i_]* = * ∑_i_p_i_ × C_i _/ P_i_* (*i* indicating covariate category).

The Poisson model–based analyses were conducted at the census tract–level using a circular spatial window that moved systematically throughout the study area to identify significant clusters with high or low rates of LCC. For the unadjusted model, the maximum cluster size was set at 25% at-risk population.^30^ A log likelihood ratio (LLR) test was used to assess the alternate hypothesis that there were elevated or reduced numbers of LCC cases within each identified SaTScan cluster compared with the distribution outside the moving SaTScan cluster. The statistical significance of identified clusters was set at 2-sided *P* < .05 and explored using 999 Monte Carlo replications to ensure adequate statistical power for defining clusters^27^ and generating their relative risks (RRs) and 95% CIs. We ran the final model adjusting for age groups at a maximum cluster set at 25% of the at-risk population. Statistically significant clusters were defined as the most likely SaTScan clusters that were least likely to have occurred by chance. We also conducted a census tract–level comparison of demographic characteristics of the SaTScan-identified hot spots vs cold spots and the rest of the state using the Wilcoxon rank-sum test comparing medians of nonparametric independent groups.

## Results

**Figure 1.**
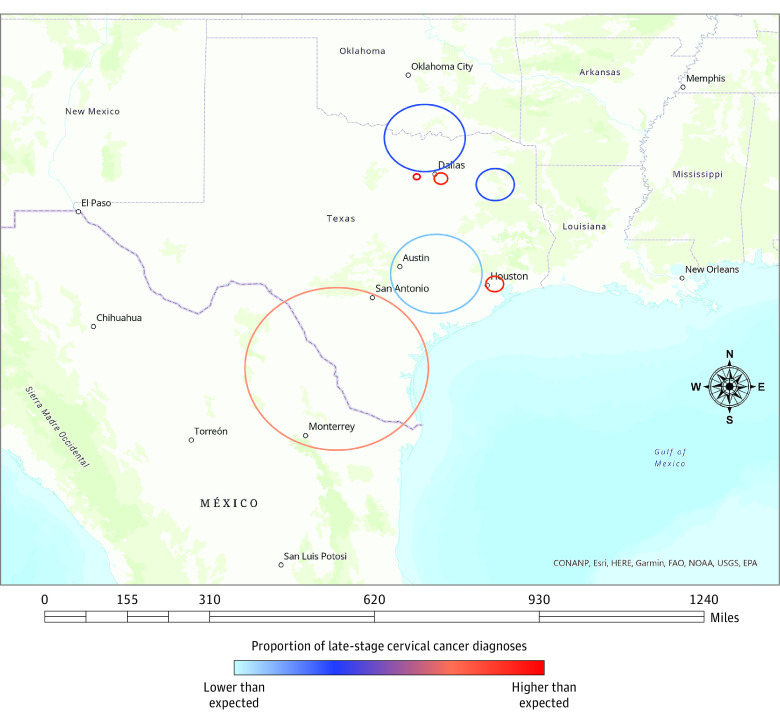
Hot Spots and Cold Spots of Late-Stage Cervical Cancer Diagnosis in Texas, 2014-2018 Geospatial map showing clusters of late-stage cervical cancer in Texas between 2014 and 2018. Blue circles represent clusters with lower-than-expected proportion of late-stage cervical cancer diagnosis (cold spots; relative risk [RR] <1), whereas red or orange circles represent clusters with higher-than-expected proportion of late-stage cervical cancer diagnosis (hot spots; RR >1). Counties within identified clusters were as follows. Cluster 1 (RR, 0.47; *P* < .001): Collin, Cooke, Dallas, Denton, Fannin, Grayson, Hunt, Montague, Tarrant, Wise; Cluster 2 (RR, 1.77; *P* < .001): Harris; Cluster 3 (RR, 0.65; *P* < .001): Austin, Bastrop, Bell, Brazos, Burleson, Caldwell, Colorado, DeWitt, Falls, Fayette, Fort Bend, Gonzales, Grimes, Guadalupe, Harris, Hays, Jackson, Lavaca, Lee, Madison, Milam, Montgomery, Robertson, Travis, Waller, Washington, Wharton, Williamson; Cluster 4 (RR, 1.42; *P* < .001): Aransas, Atascosa, Bandera, Bee, Bexar, Brooks, Cameron, Dimmit, Duval, Frio, Goliad, Hidalgo, Jim Hogg, Jim Wells, Karnes, Kenedy, Kinney, Kleberg, La Salle, Live Oak, McMullen, Maverick, Medina, Nueces, Refugio, San Patricio, Starr, Uvalde, Val Verde, Webb, Willacy, Wilson, Zapata, Zavala; Cluster 5 (RR, 2.34; *P* < .001): Tarrant; Cluster 6 (RR, 1.82; *P* = .02): Dallas, Ellis, Kaufman; Cluster 7 (RR, 0.37; *P* = .03): Cherokee, Gregg, Harrison, Henderson, Rusk, Smith, Upshur, Van Zandt, Wood.

This study analyzed 6484 female patients (mean [SD] age, 48.7 [14.7] years) with incident cervical cancer diagnosed in Texas from 2014 through 2018 and reported to the Texas Cancer Registry. Of the 6484 patients, 2300 (35.5%) were Hispanic, 798 (12.3%) were non-Hispanic Black, 3090 (47.6%) were non-Hispanic White, and 296 (4.6%) were other race or ethnicity. Among 2892 patients with an LCC diagnosis included in the study, 1069 (37.0%) were Hispanic, 417 (14.4%) were non-Hispanic Black, 1307 (45.2%) were non-Hispanic White, and 99 (3.4%) were other race; 1079 (37.3%) were publicly insured, 683 (23.6%) were uninsured, and 278 (9.6%) had unknown insurance; mean (SD) age, was 51.8 (14.4) years (Table 1). Age-adjusted Poisson-based spatial scan statistics identified a total of 7 statistically significant clusters of LCC diagnosis (Table 2), 4 of which had higher-than-expected proportions of LCC cases at diagnosis (hot spots). Hot spots included 1128 of 5265 census tracts (39 of 254 counties) in Texas. Of the 2892 patients with an LCC diagnosis, 880 (30.4%) were observed within hot spots. Hot spots were located in the South Texas Plains (near Mexico border), Gulf Coast (Houston), and Prairies and Lakes (North Texas) regions. Additional cluster details, including location and RRs, are presented in Table 2, Figure 1, and eTable in Supplement 1.

**Table 1.** Demographic Characteristics of Female Patients With CC Diagnosis in Texas, 2014-2018 and by Stage of Diagnosis^a^^,^^b^

Characteristics	Patients, No. (%)				*P* value^c^
	All CC (N = 6484)	Early-stage CC (n = 2732)	Late -stage CC (n = 2892)	Unstaged CC (n = 860)	
Age, mean (SD), y	48.7 (14.7)	44.9 (13.6)	51.8 (14.4)	49.8 (16.4)	<.001
Age group, y					
18-34	1158 (17.9)	679 (24.9)	312 (10.8)	167 (19.4)	<.001
35-64	4343 (67.0)	1782 (65.2)	2043 (70.6)	518 (60.2)	
≥65	983 (15.1)	271 (9.9)	537 (18.6)	175 (20.4)	
Race and ethnicity					
Hispanic	2300 (35.5)	951 (34.8)	1069 (37.0)	280 (32.6)	<.001
Non-Hispanic Black	798 (12.3)	273 (10.0)	417 (14.4)	108 (12.6)	
Non-Hispanic White	3090 (47.6)	1364 (49.9)	1307 (45.2)	419 (48.7)	
Other^d^	296 (4.6)	144 (5.3)	99 (3.4)	53 (6.1)	
Payer at diagnosis					
Private^e^	2398 (37.0)	1332 (48.8)	852 (29.5)	214 (24.9)	<.001
Public^f^	2043 (31.5)	693 (25.4)	1079 (37.3)	271 (31.5)	
Uninsured^g^	1237 (19.1)	392 (14.3)	683 (23.6)	162 (18.8)	
Unknown^h^	806 (12.4)	315 (11.5)	278 (9.6)	213 (24.8)	
Diagnosis year					
2014	1352 (20.9)	624 (22.9)	577 (20.0)	151 (17.5)	.08
2015	1282 (19.8)	492 (18.0)	569 (19.7)	221 (25.7)	
2016	1286 (19.8)	536 (19.6)	574 (19.8)	176 (20.5)	
2017	1258 (19.4)	533 (19.5)	592 (20.5)	133 (15.5)	
2018	1306 (20.1)	547 (20.0)	580 (20.1)	179 (20.8)	

Abbreviation: CC, incident cervical cancer.

^a^ Data retrieved from primary CC data provided by Texas Cancer Registry, 2014-2018.

^b^ Cervical cancer staging definition: early-stage (National Cancer Institute Surveillance, Epidemiology and End Results [SEER] summary stage 0 [in situ, intraepithelial, noninvasive] and 1 [localized]); late-stage SEER summary stage 2-4 (regional spread) and 7 (distant spread); unstaged SEER summary stage 9 (missing or unknown stage) cervical cancer at diagnosis.

^c^ *P* value derived from χ^2^ tests comparing characteristics of early-stage CC and late-stage CC groups for categorical variables and *t* test of difference in means for the continuous variable.

^d^ Patients with races other than White and other than Black were recoded into the Other category.

^e^ Private includes types of private insurance.

^f^ Public or government includes Medicare, Medicaid, TRICARE, military, Veteran Affairs, or Indian/Public Health Service.

^g^ Uninsured includes not insured or self-pay.

^h^ Unknown includes insurance not otherwise specified or insurance status unknown.

**Table 2.** Results of Age-Adjusted Poisson-Based Purely Spatial Scan Statistics Showing Hot and Cold Spots of Late-Stage Cervical Cancer Diagnosis in Texas, 2014-2018^a^

Cluster	Coordinate (radius, km)	Observed cases	Expected cases	Relative risk	LLR	*P* value
1	33.765773 N, 97.071925 W (101.53)	170	340.68	0.47	58.09	<.001
2	29.793699 N, 95.170742 W (23.33)	249	146.01	1.77	31.88	<.001
3	30.069870 N, 96.756974 W (119.53)	309	450.88	0.65	29.17	<.001
4	27.485009 N, 99.467114 W (245.61)	478	354.65	1.42	22.38	<.001
5	32.711811 N, 97.288611 W (8.65)	62	26.86	2.34	16.94	<.001
6	32.664184 N, 96.632680 W (17.09)	91	50.67	1.82	13.24	.02
7	32.499570 N, 95.155531 W (48.45)	19	50.50	0.37	13.10	.03
8	29.855711 N, 98.472530 W (31.56)	13	39.34	0.33	12.07	.07
9	33.657260 N, 101.687410 W (18.31)	17	3.98	4.30	11.71	.09
10	29.660619 N, 95.518253 W (6.09)	56	27.43	2.06	11.54	.09
11	29.440990 N, 95.228426 W (16.01)	10	32.88	0.30	11.07	.13
12	31.189137 N, 94.513619 W (28.39)	23	7.59	3.05	10.14	.28
13	32.757044 N, 96.943490 W (7.38)	38	17.13	2.24	9.49	.45
14	29.893117 N, 95.442305 W (4.52)	28	11.23	2.51	8.86	.65
15	29.488715 N, 95.501129 W (11.95)	6	20.78	0.29	7.37	.98

Abbreviations: LLR, log likelihood ratio; N, north; W, west.

^a^ Poisson-based purely spatial scan statistic was used to identify areas with significantly higher-than-expected proportion (hot spots) and lower-than-expected proportion of (cold spots) of late-stage cervical cancer cases across Texas. Maximum spatial cluster size was set at 25% of the population at risk, using circular scan window and replication 999.

**Figure 2.**
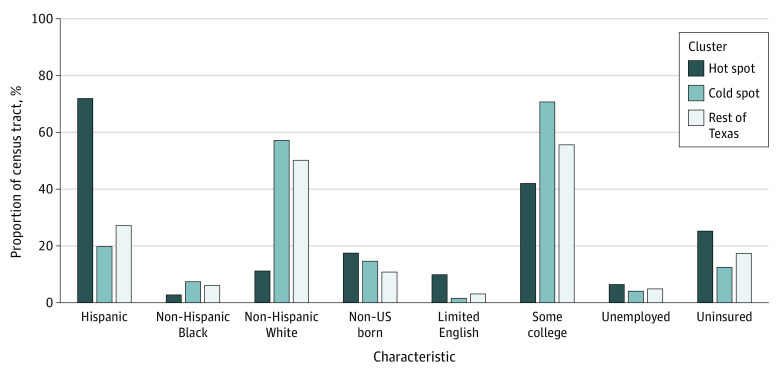
Census Tract–Level Characteristics of Patients With Late-Stage Cervical Cancer in Texas, 2014-2018 by Cluster Classification Bar chart showing the characteristics of census tracts containing late-stage cervical cancer cases stratified by cluster classification (hot spots, cold spots, rest of Texas). Census tract–level data were retrieved from the American Community Survey 5-year estimate (2014-2018). Limited English indicates Spanish-speaking households with limited English proficiency; some college, female patient aged 25 years or older with some college educational attainment; unemployed, percentage of population aged 16 years and older who are unemployed; uninsured, female patient older than 18 years with no insurance coverage.

Furthermore, a comparison of census tract–level characteristics of hot spots, cold spots, and the rest of Texas (nonclusters) found significant differences between census tracts of hot spots vs cold spots, and hot spots vs the rest of Texas in terms of racial and ethnic distribution, place of birth, socioeconomic status, and health care access. Higher proportions of Hispanic individuals, non–US born individuals, unemployed individuals aged 16 years or older, Spanish households non-English proficient, and uninsured female individuals aged 18 years or over were in hot spots compared with cold spots or the rest of Texas. In addition, hot spot census tracts had lower median household income compared with cold spots and the rest of Texas (Table 3 and Figure 2).

**Table 3.** Comparing Texas Census Tract–Level Characteristics by Cluster Classification (N = 5265)

Characteristics^a^	Late-stage cervical cancer census tracts				
	Median (IQR)			Hot spots vs cold spots *P* value^b^	Hot spots vs rest of Texas* P* value^b^
	Hot spots (n = 1128)	Cold spots (n = 1298)	Rest of Texas (n = 2839)		
Demographics					
Female median age, y	33.6 (30.3-38.3)	37.4 (32.8-42.5)	37.0 (32.3-42.7)	<.001	<.001
% Hispanic	71.9 (45.3-89.9)	19.8 (11.9-33.6)	27.2 (15.1-49.9)	<.001	<.001
% Non-Hispanic Black	2.8 (0.2-13.0)	7.4 (3.3-13.6)	6.1 (1.7-16.7)	<.001	<.001
% Non-Hispanic White	11.2 (3.9-29.1)	57.2 (38.2-71.4)	50.2 (23.3-71.2)	<.001	<.001
% Non–US born	17.5 (9.9-26.7)	14.6 (8.2-23.6)	10.8 (5.3-20.9)	<.001	<.001
% Household with limited English proficiency, Spanish	9.9 (3.6-19.8)	1.6 (0-4.7)	3.1 (0.7-8.9)	<.001	<.001
Socioeconomic status					
% Some college education female ≥25 y	42.0 (30.9-57.4)	70.7 (56.0-82.7)	55.6 (43.9-69.2)	<.001	<.001
% Unemployed ≥16 y	6.4 (4.1-9.0)	4.1 (2.8-5.8)	4.9 (3.2-7.3)	<.001	<.001
Median household income, $	42 295 (32 371-57 734)	70 997 (53 892-98 962)	52 703 (40 710-70 102)	<.001	<.001
Health care access					
% Uninsured female ≥18 y	25.2 (16.6-35.6)	12.5 (7.1-20.5)	17.4 (11.1-26.1)	<.001	<.001

^a^ Census tract–level data retrieved from the American Community Survey 5-year estimate 2014-2018 data.

^b^ Statistical significance (2-sided *P* < .05) determined using Wilcoxon rank-sum test comparing medians of nonparametric independent groups.

## Discussion

In this population-based study, using Poisson-based SaTScan spatial scan statistics, we found substantial spatial dependence in LCC at diagnosis in Texas. In other words, across Texas, we identified 4 significant hot spots with higher-than-expected proportions of LCC cases at diagnosis. Hot spots were found in the South Texas Plains (near Mexico border), Gulf Coast (Houston), and Prairies and Lakes (North Texas) regions. In addition, there were demographic and socioeconomic variations in the characteristics of hot spots census tracts compared with cold spots and the rest of Texas. Our findings are consistent with previous studies investigating geographic clusters of cervical cancer incidence, stage at diagnosis, and mortality in the US.^24,25,31,32^ For example, Roche and colleagues^24^ examined invasive cervical cancers diagnosed in New Jersey from 2005 to 2009 and found spatial disparities in the distribution of cases. The authors also reported racial and ethnic and socioeconomic differences in the characteristics of identified clusters of cases. Likewise, a previous Texas study from 1990 to 1997 reported county-level spatial clusters of cancer deaths, including cervical cancer mortality.^31^ Another study conducted from 1995 to 2008 linked geographic disparities in LCC diagnosis in Texas with race and ethnicity and neighborhood-level socioeconomic status.^25^ Hispanic and non-Hispanic Black women had greater odds of LCC diagnosis than non-Hispanic White women.^25^ Conversely, in the current study, the proportion of non-Hispanic Black female patients in hot spots was lower than in cold spots and the rest of Texas. Future cluster analyses investigating all cervical cancer diagnosis and early-stage cervical cancer are warranted to provide additional insight into race-specific and place-based variations in cervical cancer stage at diagnosis.

Texas has the largest population of uninsured nationally, and more than 4.2 million (greater than 24% of Texans in 2021) individuals 19 to 64 years of age lacked health insurance coverage (twice the national rate)^33^ and experienced worse general health status than those insured. Moreover, a larger percentage of racially and ethnically minoritized populations in the US reported an inability to see a doctor in the past year due to their inability to afford the cost of care compared with the White population.^34^ Our data showed the largest hot spot in South Texas Plains Region, including the Rio Grande Valley near Mexico border. Other hot spots were found in the Gulf Coast (Harris County, Houston) and Prairies and Lakes (North Texas) regions. The detection of hot spots in these areas may be explained by the unique population characteristics, and persistent place-based health disparities, driven by a lack of access to health care and socioeconomic disadvantages. Moreover, proximity and availability of health care resources may not equal access, particularly in large cities that have health care resources that are just inaccessible due to high cost.^34,35^

Harris County, for example, is the third largest county in the US, with the largest racial/ethnic group being Hispanic (44.6%)^36^ Likewise, other hot spots identified in this study have Hispanic majority populations compared with other areas in the US. For instance, South Texas Plains is predominantly Hispanic (84%).^37^ Additionally, many of the hot spot census tracts have a higher than the national proportion of populations who are non–US born, uninsured, and with low socioeconomic status.^36^ Low income, uninsured, and non–US born individuals with reduced access to cervical cancer prevention may not prioritize preventive services utilization, thus present with LCC. In addition, other social and environmental stressors, such as racism, immigration status, language barriers, and neighborhood-level socioeconomic disadvantage, may undermine health care access, including cervical cancer screening.

Therefore, LCC geographic disparities in Texas may be related to higher percentages of racial minority populations and socioeconomic disadvantage prevalent across the identified hot spot areas compared to other areas in Texas. Studies have suggested that rates of LCC diagnosis are rising, and the South had the greatest annual increase from 2001 to 2018.^7^

Cervical cancer is almost entirely preventable through vaccination against HPV and early detection and treatment of precancerous lesions. Clusters of LCC likely indicate systemic missed opportunities for prevention. Many challenges to cervical cancer equity require scaled up and equitable access to preventive services, including HPV vaccination and screening, as well as follow-up treatment. This is particularly challenging in Texas, a state that is yet to expand Medicaid coverage. Intervention is needed throughout Texas to reduce the disproportionate morbidity and mortality burden from the disease. Specifically, the 4 geographic hot spots with significantly higher risks of delayed cervical cancer diagnosis need strategic and aggressive interventions for cervical cancer prevention, including culturally sensitive education, vaccination, and screening. Screening is particularly an urgent strategy given that there are several generations of persons at risk for cervical cancer who did not receive the HPV vaccine earlier in life. Indeed, modeling studies indicate that scaling up screening can expedite efforts to control the public health burden of cervical cancer.^38^ In 2020, the American Cancer Society updated its screening recommendation to primary HPV testing as the preferred screening test for cervical cancer screening.^39^ HPV self-sampling is an effective and efficient cervical cancer screening method targeted at underscreened women in many countries globally.^40^ It is expedient to examine innovative interventions that address multiple barriers to cervical cancer screening in these areas, such as HPV self-sampling. Additionally, future research should test the feasibility and effectiveness of opportunistic cervical cancer interventions, including culturally sensitive and evidenced-informed education, vaccination, and HPV self-sampling in schools, communities, clinics, and pharmacies in these locations.

### Limitations

The results of this study should be interpreted within the context of its limitations. First, TCR does not provide data on past cervical cancer screening, which has a major influence on cancer stage at diagnosis.^41^ Second, we cannot rule out misclassification bias because our data did not include cases retrieved from death certificates, or missing stage information. This may result in missing other potential hot spots of LCC.^42^ Third, it is possible that race and ethnicity were misclassified during data collection. Procedures for assigning and verifying codes for race and ethnicity are not well standardized.^43^ Fourth, data analyses could not be conducted at smaller geographic units beyond the census tracts, because cervical cancer (especially LCC) is a rare disease, hence inferences cannot be made beyond this unit. Additionally, the possibility of ecological fallacy cannot be excluded because our investigation was conducted at area level and may not be attributable to individuals.^44^ Despite limitations, the findings of this study are notable and consistent with previous studies that have documented Texas geographic and racial and ethnic disparities in cervical cancer stage at diagnosis.^25^ Also, this study is based on large population-based data from an NCI SEER program cancer registry that meets the high-quality standards. Furthermore, SaTScan spatial analysis used in identifying geographic clusters is a free globally used cluster-detecting epidemiologic tool for identifying spatial clusters of infectious and chronic diseases, including COVID-19, malaria, cancers, and others.^24,45,46,47,48,49,50,51,52,53,54^

## Conclusions

In this cross-sectional study of spatial clusters of LCC, we found significant geographic variation in LCC diagnosis in Texas. We identified 4 hot spots (clusters with higher-than-expected proportion of cases) of LCC diagnosis in South Texas Plains (near Mexico border), Gulf Coast (Houston), and Prairies and Lakes (North Texas) regions, with mostly higher than national proportions of Hispanic populations and socioeconomic disadvantage. These areas can be specifically targeted for aggressive cervical cancer interventions, including education, vaccination, and screening.

## Notes

Supplement 1. **eTable.** Statistically Significant Clusters of Late-Stage Cervical Cancer Diagnosis in Texas, 2014-2018

Supplement 2.
Data Sharing Statement
