# Supplementary material for: Geographic Variation in Late-Stage Cervical Cancer Diagnosis
Source: JAMA Netw Open. 2023 Nov 13;6(11):e2343152. doi: 10.1001/jamanetworkopen.2023.43152 (PMC10644213; doi:10.1001/jamanetworkopen.2023.43152)
Supplement: Supplement 1. — eTable. Statistically Significant Clusters of Late-Stage Cervical Cancer Diagnosis in Texas, 2014-2018 [file jamanetwopen-e2343152-s001.pdf]

Supplemental Online Content

Sokale IO, Thrift AP, Montealegre J, et al. Geographic variation in late-stage cervical cancer diagnosis in Texas. *JAMA Netw Open.* 2023;6(11):e2343152. doi:10.1001/jamanetworkopen.2023.43152

eTable. Statistically Significant Clusters of Late-Stage Cervical Cancer Diagnosis in Texas, 2014-2018

This supplemental material has been provided by the authors to give readers additional information about their work.

© 2023 Sokale IOet al. *JAMA Network Open.*

eTable. Statistically significant clusters of late-stage cervical cancer diagnosis in Texas, 2014-2018[a]

Cluster	County	No. of census tracts within clusters per county (%)	Total no. of census tracts in the county
**1 – Cold Spot** (RR=0.47)	Collin	150 (99)	152
	Cooke	8 (100)	8
	Dallas	143 (27)	529
	Denton	137 (100)	137
	Fannin	7 (78)	9
	Grayson	26 (100)	26
	Hunt	1 (5)	19
	Montague	6 (100)	6
	Tarrant	60 (17)	357
	Wise	10 (91)	11
**2 – Hot Spot** (RR=1.77)	Harris	295 (38)	786
**3 – Cold Spot** (RR=0.65)	Austin	6 (100)	6
	Bastrop	10 (100)	10
	Bell	2 (3)	65
	Brazos	42 (100)	42
	Burleson	5 (100)	5
	Caldwell	8 (100)	8
	Colorado	5 (100)	5
	DeWitt	3 (60)	5
	Falls	1 (17)	6
	Fayette	7 (100)	7
	Fort Bend	38 (50)	76
	Gonzales	6 (100)	6
	Grimes	6 (100)	6
	Guadalupe	3 (10)	29
	Harris	152 (19)	786
	Hays	16 (64)	25
	Jackson	1 (33)	3
	Lavaca	6 (100)	6
	Lee	4 (100)	4
	Madison	1 (25)	4
	Milam	7 (100)	7
	Montgomery	14 (24)	59
	Robertson	4 (80)	5
	Travis	210 (96)	218
	Waller	6 (100)	6
	Washington	6 (100)	6
	Wharton	11 (100)	11
	Williamson	82 (92)	89

© 2023 Sokale IOet al. *JAMA Network Open.*

eTable (continued). Statistically significant clusters of late-stage cervical cancer diagnosis in Texas, 2014-2018[a]

Cluster	County	No. of census tracts within clusters per county (%)	Total no. of census tracts in the county
**4 – Hot Spot** (RR=1.42)	Aransas	3 (50)	6
	Atascosa	8 (100)	8
	Bandera	1 (20)	5
	Bee	7 (100)	7
	Bexar	253 (69)	366
	Brooks	2 (100)	2
	Cameron	33 (38)	87
	Dimmit	2 (100)	2
	Duval	3 (100)	3
	Frio	3 (100)	3
	Goliad	1 (50)	2
	Hidalgo	113 (100)	113
	Jim Hogg	2 (100)	2
	Jim Wells	7 (100)	7
	Karnes	4 (100)	4
	Kenedy	2 (100)	2
	Kinney	1 (100)	1
	Kleberg	6 (100)	6
	La Salle	1 (100)	1
	Live Oak	4 (100)	4
	McMullen	1 (100)	1
	Maverick	9 (100)	9
	Medina	8 (100)	8
	Nueces	82 (100)	82
	Refugio	2 (100)	2
	San Patricio	16 (100)	16
	Starr	15 (100)	15
	Uvalde	5 (100)	5
	Val Verde	1 (10)	10
	Webb	61 (100)	61
	Willacy	5 (83)	6
	Wilson	10 (91)	11
	Zapata	3 (100)	3
	Zavala	4 (100)	4
**5 – Hot Spot** (RR=2.34)	Tarrant	68 (19)	357
**6 – Hot Spot** (RR=1.82)	Dallas	84 (16)	529
	Ellis	1 (3)	31
	Kaufman	2 (11)	18
**7 – Cold Spot** (RR=0.37)	Cherokee	1 (8)	12
	Gregg	25 (100)	25
	Harrison	1 (7)	14
	Henderson	1 (6)	16
	Rusk	5 (38)	13
	Smith	41 (100)	41
	Upshur	6 (86)	7
	Van Zandt	1 (11)	9
	Wood	7 (70)	10

Abbreviations: RR, relative risk.
[a]Data was retrieved from the age-adjusted Poisson-based purely spatial scan statistic using the American Community survey 5-year estimate data (2014-2018) and late-stage cervical cancer (LCC) reported to Texas Cancer Registry from 2014-2018. Hot spots are clusters with significantly higher than expected proportion of LCC diagnosis, while cold spots are clusters with significantly lower

© 2023 Sokale IOet al. *JAMA Network Open.*

than expected proportion of LCC diagnosis. Maximum spatial cluster size was set at 25 percent of the population at risk, using circular scan window and Replication 999.

© 2023 Sokale IOet al. *JAMA Network Open.*
