# Supplementary material for: Geographic Variation in Late-Stage Cervical Cancer Diagnosis
Source: JAMA Netw Open. 2023 Nov 13;6(11):e2343152. doi: 10.1001/jamanetworkopen.2023.43152 (PMC10644213; doi:10.1001/jamanetworkopen.2023.43152)
Supplement: Supplement 2. — Data Sharing Statement [file jamanetwopen-e2343152-s002.pdf]

Data Sharing Statement

Sokale. Geographic Variation in Late-Stage Cervical Cancer Diagnosis in Texas. *JAMA Netw Open*. Published November 13, 2023. doi:10.1001/jamanetworkopen.2023.43152

Data
Data available: No
